# Study on the Properties of Multi-Layer Cumulative Rolling-Prepared High-Chromium Cast Iron Powder/Low-Carbon Steel Composites

**DOI:** 10.3390/ma19050839

**Published:** 2026-02-24

**Authors:** Yulin Xing, Wenbo Gao, Xiaogang Wang, Yunlong Zhu, Mantang Yu

**Affiliations:** State Key Laboratory of Advanced Stainless Steel, Taiyuan University of Science and Technology, Taiyuan 030024, China; b202247110041@stu.tyust.edu.cn (Y.X.); s202447211300@stu.tyust.edu.cn (W.G.); s202347211299@stu.tyust.edu.cn (Y.Z.); yumantang@tyust.edu.cn (M.Y.)

**Keywords:** hot-rolled forming, composite materials, microstructure, high-chromium cast iron powder

## Abstract

**Highlights:**

**What are the main findings?**

- Multi-pass hot rolling (with a total thickness reduction of 70%) was successfully employed to fabricate high-chromium cast iron (HCCI) powder/low-carbon steel multilayer composites.
- The tensile strength exhibited a non-monotonic variation with temperature, peaking at approximately 810 MPa at 1150 °C. The composite showed very limited macroscopic plasticity and underwent brittle fracture, with cracks initiating in the HCCI layer.
- A rolling temperature of 1150 °C was identified as optimal, offering the best combination of mechanical properties and interfacial bonding.

**What are the implications of the main findings?**

- This work provides benchmark parameters for the development of similar bimetallic wear-resistant composites.
- The study demonstrates a low-cost and feasible route for producing wear-resistant composites via hot rolling of coarse powder.
- It also offers a valuable research paradigm for the study of other powder metallurgy-based laminated composites.

**Abstract:**

Multilayer laminated composites consisting of high-chromium cast iron (HCCI) powder clad with low-carbon steel (LCS) were fabricated via multi-pass hot rolling at a deformation of 70% under three different temperatures: 1100 °C, 1150 °C, and 1200 °C. The microstructure, elemental diffusion, and mechanical properties of the samples processed at these temperatures were systematically investigated. The results indicate that effective metallurgical bonding was achieved between the HCCI powder and the LCS matrix, with the HCCI regions accumulating high strain energy and dislocation density. Hardness testing demonstrated that higher rolling temperatures lead to increased hardness. The dominant wear mechanism was identified as dry sliding wear. The relatively low content of retained austenite contributed to a reduction in tensile strength, while this microstructure further promoted abrasive wear through the spalling of carbides. These findings suggest that hot processing offers a feasible pathway for improving the wear resistance of HCCI-based composites.

## 1. Introduction

Bimetallic wear-resistant composites typically consist of a tough substrate, such as low-carbon steel, low-alloy steel, or stainless steel, designed to withstand applied loads, paired with a high-hardness wear-resistant layer to resist abrasion [1,2]. High-chromium cast iron (HCCI), characterized by its high chromium content, is a quintessential chromium-based white cast iron. Recognized as a third-generation wear-resistant material, it serves as an outstanding wear-resistant alloy due to its superior wear resistance compared to alloy steels of similar hardness. Consequently, HCCI is extensively used in wear-resistant components within industries such as metallurgy and mining [3,4,5]. However, in practical applications, the high volume fraction of carbides in HCCI can lead to fracture and failure under impact, hindering the full utilization of its excellent wear resistance. Therefore, for HCCI, an outstanding wear-resistant metal, integrating it with ductile materials to form bimetallic composites, thereby enhancing overall mechanical properties and enabling broader application, represents an urgent issue requiring resolution.

Due to the presence of internal carbides, high-chromium cast iron exhibits high hardness. Jaramillo, R et al. [6], through their study on the influence of varying Cr content on carbide hardness, discovered that wear resistance increases as Cr content rises. Various methods are currently employed to prepare high-chromium cast iron/low-carbon steel (HCCI/LCS) bimetallic composite plates, including casting [7,8,9,10,11], surfacing [12,13], casting–hot rolling [14,15], hot-press diffusion bonding [16,17,18], and powder metallurgy [19]. Eroglu et al. [12] bonded the two materials via diffusion and found that increasing the diffusion temperature and holding time effectively enhanced the joint strength. Javaheri et al. [13] prepared carbon steel/HCCI bimetallic composites using a liquid–solid casting method, reporting excellent metallurgical bonding with a pearlite diffusion zone at the interface. Performance tests indicated that the elongation and impact toughness of the bimetallic material, while lower than those of plain carbon steel, were significantly superior to those of monolithic HCCI plate. Sallam et al. [9] utilized casting technology to prepare a composite from HCCI and AISI 4140 steel containing 19% chromium.

The rolling process is also an effective route for preparing bimetallic composites. Composites produced this way achieve sound metallurgical bonding between dissimilar metals, and their microstructure can be further improved through elemental interdiffusion. Existing research shows that the cast-rolling method is commonly used to fabricate HCCI/LCS bimetallic composites. For instance, Liu et al. [15] manufactured a sandwich-structured composite tube via centrifugal casting, followed by hot rolling of the billet at 1170 °C to produce a bimetallic plate. Sakurai et al. [16] experimentally studied the diffusion bonding of HCCI and low-carbon steel at temperatures ranging from 1173 K to 1273 K, under varying diffusion times and pressures in an unprotected atmosphere. Their findings indicate that optimal interfacial bond strength was achieved at diffusion temperatures of 1223–1273 K, a bonding time of 10.8 ks, and an applied pressure of 9.8 MPa. Yuan et al. [20] developed a hot compression process to prepare a high-chromium cast iron/low-carbon steel composite, which exhibited good coordinated deformation ability at 1150 °C.

Inspired by prior research, we explored a solid-state processing route to prepare HCCI/LCS bimetallic composites via vacuum hot-rolling. Multi-layer stack rolling was employed to fully leverage the advantages of both constituent materials and enhance the composite’s overall performance. In this study, multilayer composites were successfully fabricated by hot rolling, using powdered high-chromium cast iron encapsulated within low-carbon steel as a substitute for lump HCCI. The microstructure at the interfaces after hot-rolling bonding under 70% deformation was investigated using scanning electron microscopy (SEM). Additionally, the mechanical properties of the composite plates were evaluated and analyzed through tensile and friction-wear testing.

## 2. Materials and Methods

### 2.1. Material Preparation

**Figure 1:**
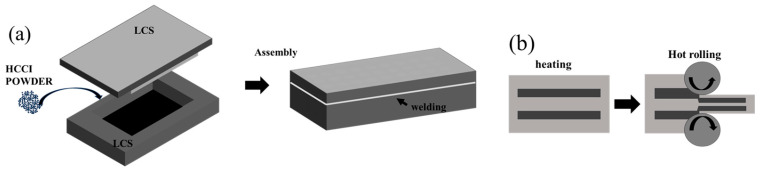
Schematic Diagram of Experimental Group Blanks (**a**) Blanking Process; (**b**) Rolling Process.

HCCI and Q235 LCS were used as the constituent materials. The billet preparation involved the following steps as shown in Figure 1: First, an 80 mm × 60 mm × 4 mm recess was machined into a 100 mm × 80 mm × 6 mm LCS plate to serve as a lower mold. Subsequently, a 20 mm × 1 mm peripheral strip was removed from a 100 mm × 80 mm × 2 mm LCS plate to create a matching upper mold. The resulting cavity was filled with HCCI powder, which was compacted using a press and stacked in three layers. The assembly was then evacuated, and the side seams were welded to ensure an airtight seal. The chemical compositions of the materials are listed in Table 1.

The sealed billets were heated in a medium-frequency induction furnace to three different temperatures: 1100 °C, 1150 °C, and 1200 °C. They were held at these temperatures for 30 min for homogenization before undergoing multi-pass hot rolling on a two-roll mill. The rolling speed was maintained at 0.1 m/s, with a total thickness reduction of 70% achieved. The reduction per pass was carefully controlled not to exceed 15% to avoid excessive strain per step. After rolling, the composite plates were air-cooled to room temperature. Finally, metallographic, friction-wear, and tensile specimens were sectioned from the plates using wire electrical discharge machining, following the relevant national standards.

### 2.2. Mechanical and Tribological Properties Testing

**Figure 2:**
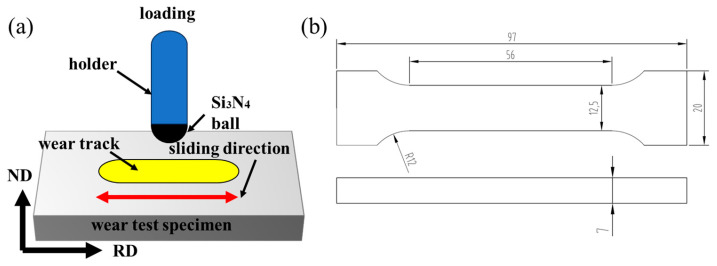
(**a**) Friction and Wear Test; (**b**) tensile specimens.

Tensile Test: Dog-bone-shaped flat tensile specimens were machined along the rolling direction (RD) of the composite plate. The geometry and sampling schematic are shown in Figure 2b. The gauge length was 56 mm, the width was 12.5 mm, and the thickness was equal to the total thickness of the composite plate (approximately 7 mm). Tests were conducted on a universal testing machine under displacement control with a crosshead speed of 0.5 mm/min. It should be noted that this test was primarily designed to evaluate and compare the fracture strength and relative deformation behavior of composites under different processing conditions. Due to the constraints of specimen size and gripping configuration, an extensometer was not used to directly measure the true strain within the gauge section. Therefore, the stress reported in the text is the true stress converted from engineering stress using the relevant formula; the strain (displacement) is the true strain converted from engineering strain using the relevant formula. For each composite plate prepared under a given rolling parameter, a minimum of three valid specimens were fabricated and tested. The results are presented as the mean value with standard deviation reported. For each composite plate produced under a given rolling parameter, a minimum of three valid specimens were tested. The results are presented as the mean value.

Hardness Test: The hardness distribution across the cross-section of the composite plate was measured using a Vickers microhardness tester (Laizhou Laishuo Test Instrument Co., Ltd., ZHVS-5Abem, Laizhou, China). A test load of 500 g was applied with a dwell time of 10 s. Measurements were taken along a straight line perpendicular to the interface, starting from the bonding surface between the two materials and extending through both the left and right low-carbon steel sides as well as the high-chromium cast iron powder side, with a step spacing of 50 μm. At least 20 valid data points were collected from each region for statistical analysis Using Origin 2018 to process tensile and hardness data.

Tribological Properties Testing: Dry sliding wear tests were conducted on a reciprocating RTec MFT-5000 tribometer (RTec, Nanjing, China). A 6 mm diameter Si_3_N_4_ ball was used as the counterface under a constant normal load of 140 N. Tests were performed for 0.5 h at a sliding frequency of 1 Hz. Post-test, the wear scar profiles were measured and analyzed using a VHX-2000C ultra-high-resolution 3D microscope (Keyence, Osaka, Japan).

Quasi-static tensile tests were performed on the rolled composites using an AGS-X electronic universal testing machine (Shimadzu, Kyoto, Japan) at a constant crosshead speed of 1 mm/min.

### 2.3. Microstructural Observation

Microstructural observation of the composite plate was carried out using a Keyence VHX-2000 deep-depth three-dimensional microscope (Keyence, Osaka, Japan). The sample was ground and polished successively with 80#, 240#, 500# up to 2000# abrasive papers, rotating the sample by 90° after each paper change. The low-carbon steel region was etched with 4% nitric acid alcohol, while the high-chromium cast iron region was etched with a hydrochloric acid-ferric chloride solution (5% FeCl_3_, 25% HCl, 70% distilled water). Vickers microhardness of the low-carbon steel, interface region, and high-chromium cast iron was measured using a Vickers hardness tester under a load of 0.5 kg with a dwell time of 15 s. Points were selected every 20 μm from the interface toward both sides, with three measurements taken at each point and averaged. A ZEISS SEM (ZEISS, Oberkochen, Germany) equipped with an Aztec EDS system was employed to analyze the microstructural evolution, fracture surfaces, and interfacial characteristics. Elemental distribution across the interfaces was investigated via energy-dispersive X-ray spectroscopy (EDS). Electron backscatter diffraction (EBSD) analysis was conducted with the sample tilted at 70°, using a step size of 0.5 µm. Acquired data were processed with OIM Analysis™ software. For grain boundary characterization, boundaries with misorientation angles below 15° were defined as low-angle grain boundaries (LAGBs), while those above 15° were classified as high-angle grain boundaries (HAGBs).

## 3. Results and Conclusions

### 3.1. Deformation of High-Chromium Cast Iron Powder Inside the Sleeve

**Figure 3:**
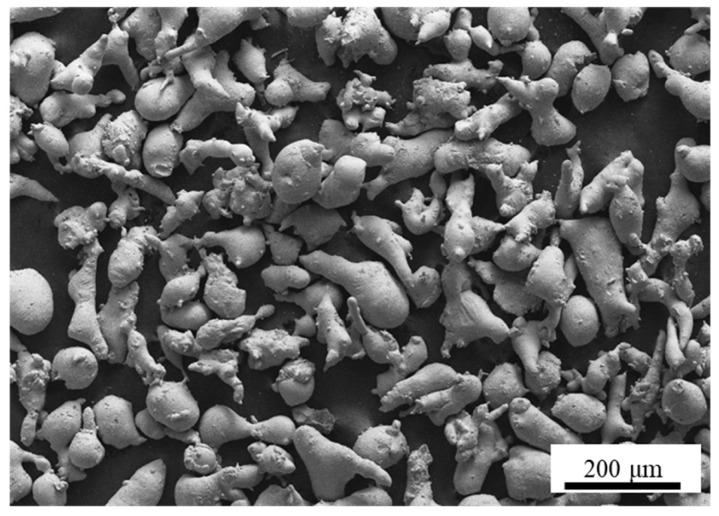
SEM image of the high-chromium cast iron in powder form.

Figure 3 presents the scanning electron microscope (SEM) morphology of the high-chromium cast iron powder used in the experiment. It can be observed that the alloy powder exhibits an overall irregular geometric shape, with good dispersion between particles and no significant agglomeration, indicating excellent flowability.

**Figure 4:**
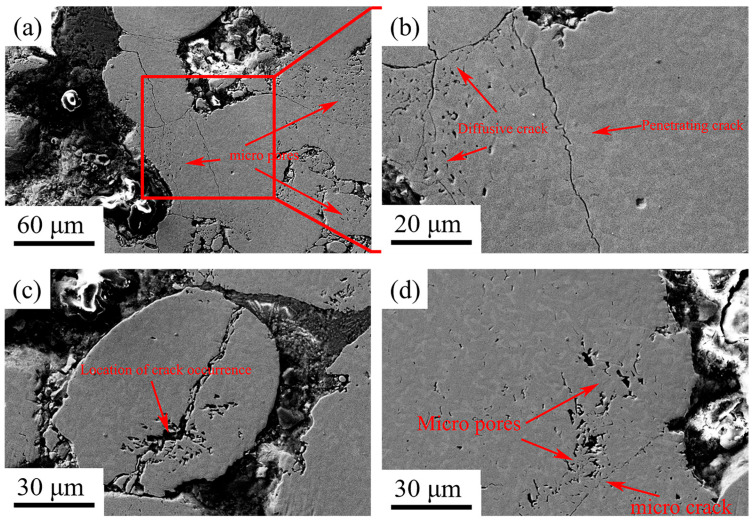
SEM image of the high-chromium cast iron powder region at 1100 °C showing deformation. (**a**) grain fragmentation, (**b**) macro-grain cracks, (**c**) entire particle fracture, (**d**) internal micropores within particles.

During hot rolling, the encapsulated HCCI powder particles, given their inherent high hardness and brittleness, primarily undergo brittle fracture. As illustrated in Figure 4, this fracture process is typically stress-concentration driven, beginning with microcrack initiation. Under applied pressure, point contacts between particles or between particles and the ductile LCS matrix lead to sharply elevated local stresses. These stresses first concentrate at pre-existing defects within the particles, such as inherent porosity or, more critically, at the interfaces between the extremely hard M_7_C_3_ carbides and the relatively softer metallic matrix, a classic weak point in HCCI. Once the local stress surpasses the material’s strength limit, microcracks nucleate. Subsequent rapid crack propagation follows the path of least resistance, often resulting in cleavage or intergranular fracture. Consequently, the initially irregular powder particles are crushed and refined into smaller, flattened fragments, achieving densification and mechanical interlocking with the surrounding matrix. Furthermore, as seen in Figure 4d, numerous fine secondary cracks branching from the primary fracture path are observed. Under complex stress states, such crack bifurcation is common, leading to the formation of these secondary cracks.

When powder particles are subjected to pressure within a ductile casing, fracture occurs. At this point, the ductile casing material, being in a high-temperature state, exhibits a degree of fluidity. This aids in forming a new, interlocked composite architecture. Moreover, during rolling, friction between the powder and the cladding, as well as inter-particle friction, leads to an uneven distribution of rolling force within the powder bed. This inhomogeneity causes pressure gradients, which is a primary factor behind the observed density variations and the initiation of cracks within the consolidated powder region.

### 3.2. Diffusion Behavior at Interface Elements

**Figure 5:**
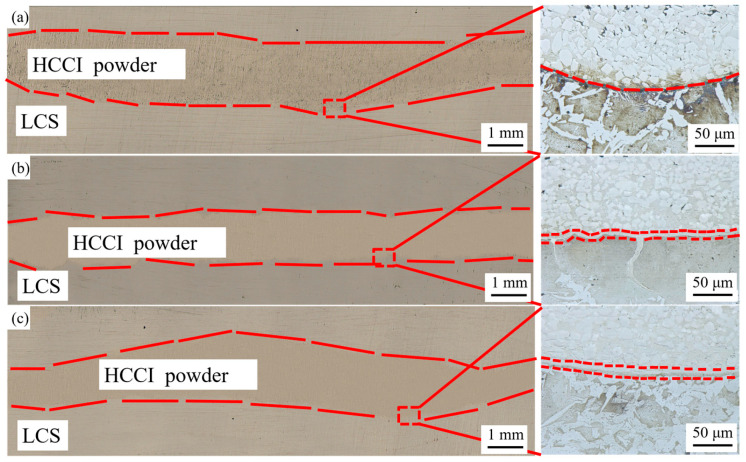
Macroscopic view and microstructure at the interface of the composite material: (**a**) 1100 °C, (**b**) 1150 °C, and (**c**) 1200 °C.

As shown in Figure 5, the macroscopic morphology of the rolled sheet exhibits good overall bonding with a continuous and clear interface. It can be observed from the figure that as the deformation temperature increases, the flowability of the high-chromium cast iron powder improves, and the porosity within the high-chromium cast iron region gradually decreases. When the deformation temperature reaches 1150 °C, part of the high-chromium cast iron powder penetrates into the low-carbon steel matrix, forming a dendritic interlocking structure. With further increase in deformation temperature, a carbide-free zone appears near the high-chromium cast iron side of the bonding interface.

**Figure 6:**
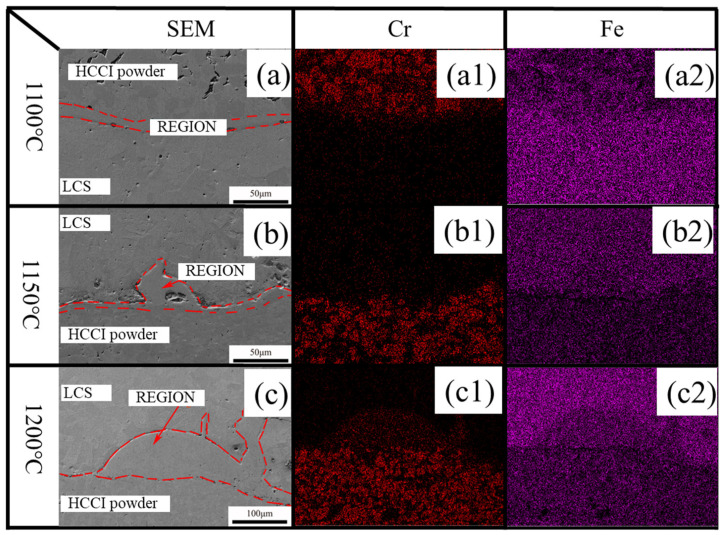
Element diffusion phenomena at the interface observed by scanning electron microscopy at: (**a**) 1100 °C, (**a1**) Cr element, (**a2**) Fe element; (**b**) 1150 °C, (**b1**) Cr element, (**b2**) Fe element; (**c**) 1200 °C, (**c1**) Cr element, (**c2**) Fe element.

Figure 6 presents EDS analysis results across the bonding interface along the Rolling Direction (RD) and Normal Direction (ND). The distinct banded contrast at the interface confirms successful metallurgical fusion between the two metals. On the HCCI side, the presence of microcracks is evident, with both eutectic and primary carbides fractured into fine fragments. This fracture of chromium carbides during hot rolling effectively lowers the deformation resistance of the HCCI matrix. SEM micrographs in Figure 6a–c characterize the interface for samples processed at 1100 °C, 1150 °C, and 1200 °C, respectively. A visible transition zone forms adjacent to the interface, where the refined, fragmented carbides contribute to enhancing the overall toughness of the high-chromium cast iron region [15].

Furthermore, EDS line scanning reveals the relative chromium content across the interface. Interdiffusion of alloying elements and continuous microstructural evolution have facilitated a sound metallurgical bond between the HCCI powder and the LCS matrix, indicative of high interfacial strength. The relatively narrow width of the diffusion zone also helps in preserving a high Cr content within the HCCI region, which is crucial for maintaining its wear resistance.

### 3.3. Research and Analysis of Friction and Wear Properties

**Figure 7:**
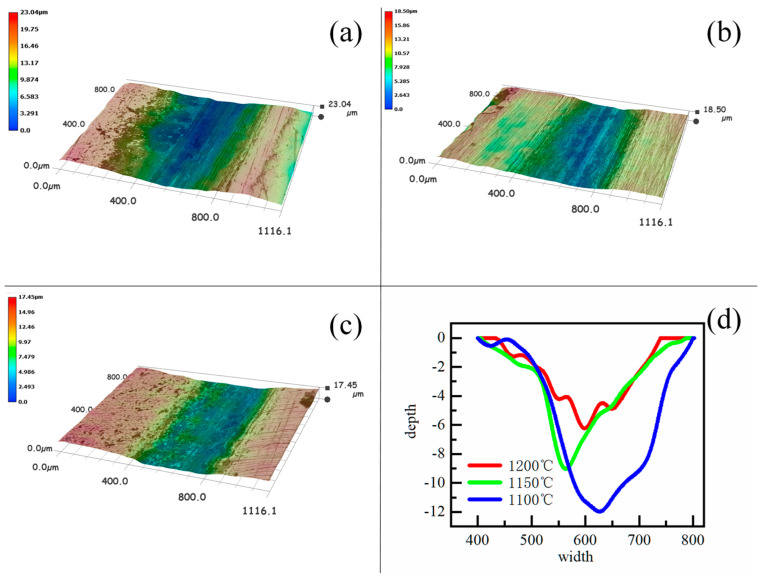
Width morphology and depth of friction wear grooves: (**a**) 1100 °C; (**b**) 1150 °C; (**c**) 1200 °C. (**d**) Groove profile.

Friction and wear tests were performed on the rolled composite plates. The worn surface topography was examined and analyzed using the 3D imaging capability of a super-depth-of-field microscope. Figure 7 shows the morphology of the wear scars obtained under a normal load of 140 N. Since the counterface was a Si_3_N_4_ sphere, the cross-sectional profile of the wear tracks exhibits a curved, arcuate shape. Under identical testing conditions, distinct differences are observed in the width and depth of the wear scars, reflecting the varying wear resistance of specimens processed under different rolling temperatures. For the sample rolled at 1100 °C, the wear track width was approximately 400 ± 0.5 μm. As seen in Figure 7a–d, both the width and depth of the wear scar for the 1100 °C specimen are significantly greater than those for specimens processed at higher temperatures. Figure 7c shows the groove at 1200 °C.

**Figure 8:**
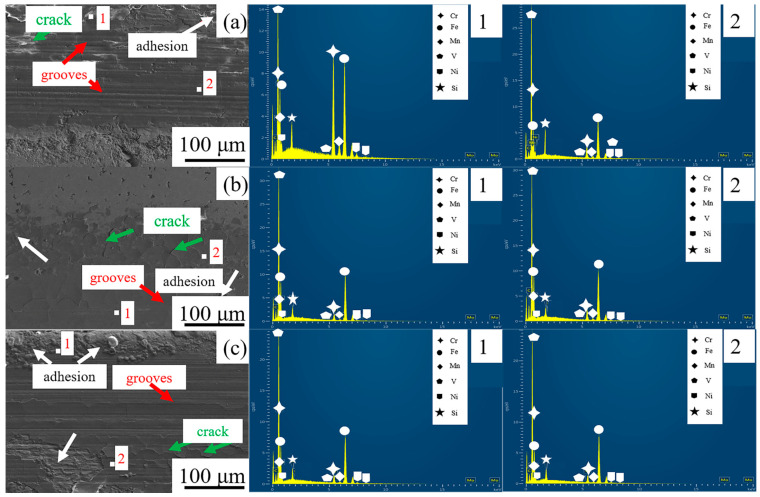
SEM images and corresponding EDS analysis of selected regions on the worn surfaces for samples rolled at (**a**) 1100 °C, (**b**) 1150 °C, and (**c**) 1200 °C. Arrows indicate specific features: green for cracks, white for adhesive zones, and red for abrasive grooves. 1 and 2 refer to the wear scar edge and the wear scar area, respectively.

Sliding wear analysis of the composites rolled at different temperatures was performed to correlate the surface morphology with the underlying wear mechanisms. Key aspects investigated included crack propagation, debris (chip) distribution, and compositional changes within the wear scars. Distinct wear patterns were observed under all three processing conditions. The composite rolled at 1200 °C (Figure 8c) demonstrated the best wear resistance. Its worn surface exhibited only shallow, parallel grooves and minor adhesive zones, with plastic deformation evident as ploughing marks. Energy-dispersive X-ray spectroscopy (EDS) revealed an increased silicon (Si) content (~1.79 wt.%) on this surface, attributable to material transfer from the Si_3_N_4_ counterface. These characteristics suggest that the primary wear mechanism at 1200 °C is adhesive wear, with a negligible contribution from oxidation. In contrast, severe surface degradation occurred in the composite rolled at 1100 °C (Figure 8a). The wear scar featured deep grooves, channel-like depressions, extensive debris accumulation, and pronounced crack propagation at the interface. The damaged surface consisted of large agglomerates of fragmented material containing numerous pits. EDS point analysis confirmed the presence of oxygen-rich fragments, while adhesive grooves were dominant in brighter regions. Consequently, the wear mechanism at 1100 °C involves a combination of oxidation fatigue, adhesion, and abrasion.

For the composite processed at 1150 °C (Figure 8b), the wear surface indicated a mechanism dominated by abrasion, with limited adhesive wear. A distinct fish-scale pattern, oriented perpendicular to the rolling direction, was also observed. Notably, the sample rolled at 1100 °C showed the most severe damage, including vertical cracks and extensive spalling. These observations collectively indicate a clear trend: wear resistance deteriorates progressively as the rolling temperature decreases, with optimal performance at 1200 °C and the poorest at 1100 °C. The enhanced wear resistance at higher temperatures is attributed to improved metallurgical bonding between the HCCI powder and the LCS matrix, facilitated by more extensive elemental interdiffusion. At lower temperatures (e.g., 1100 °C), weaker interfacial bonding and insufficient diffusion lead to the decohesion and shedding of powder particles under high contact stress, accelerating composite wear.

**Figure 9:**
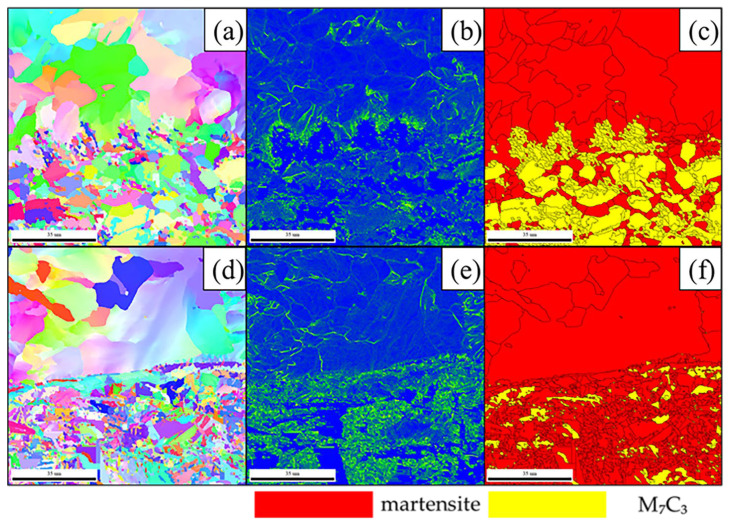
EBSD analysis of the composite: (**a**,**d**)—inverse pole figure (IPF); (**b**,**c**)—kernel average misorientation (KAM) map; (**c**,**f**)—phase map (**a**–**c**—1100 °C; **d**–**f**—1200 °C).

As shown in phase diagrams (Figure 9a,d), carbon diffusion from the HCCI powder promotes the formation of a pearlitic structure in the near-interface region of the LCS. Meanwhile, the HCCI powder itself maintains a martensitic matrix with dispersed carbides. A clear divergence in grain size evolution is observed across the interface with increasing rolling temperature. In the LCS region, grain size shows a positive correlation with temperature, whereas the grain size within the HCCI powder region exhibits an inverse relationship, becoming finer at higher temperatures. At 1200 °C, a distinct band of pearlite is visible adjacent to the HCCI side, as marked in Figure 9d.

KAM analysis is an established technique for assessing stored strain energy, which correlates directly with dislocation density [21,22]. The EBSD-derived KAM maps in Figure 9b,e visualize the distribution of residual strain and dislocations, with more intense coloration indicating higher local plastic deformation and dislocation density. Analysis shows that martensitic regions, especially in the specimen rolled at 1200 °C, exhibit significantly higher KAM values, reflecting greater dislocation accumulation. In contrast, the sample processed at 1100 °C displays a lower and more uniform KAM distribution.

In the phase maps (Figure 9c,f), red represents the martensitic matrix and yellow denotes M_7_C_3_ carbides. After hot rolling, the austenite and martensite phases within the high-chromium cast iron matrix form the primary deformation zones. Quantitative analysis reveals a clear temperature dependence of the M_7_C_3_ carbide volume fraction. Specifically, after rolling at 1100 °C, the sample contains a higher volume fraction of martensite and a correspondingly lower fraction of carbides compared to those processed at higher temperatures.

**Figure 10:**
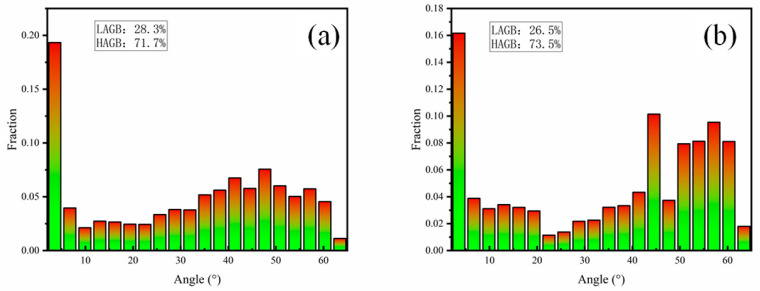
Large-angle grain boundaries in the composite material after rolling at different temperatures: (**a**) 1100 °C; (**b**) 1200 °C.

A significant difference in grain boundary character is observed between the two processing temperatures. The composite rolled at 1200 °C (Figure 10b) possesses a notably higher proportion of HAGBs at 73.5%, with only 26.5% LAGBs. This high HAGB fraction suggests extensive recrystallization or grain growth, resulting in limited residual strain. Conversely, the material rolled at 1100 °C (Figure 10a) exhibits a higher fraction of LAGBs (28.7%), indicative of greater retained lattice distortion and stored strain energy from deformation. Collectively, these results underscore the profound influence of deformation temperature on the crystallographic texture, interfacial evolution, and final phase distribution within the composite.

### 3.4. Study on Mechanical Properties

**Figure 11:**
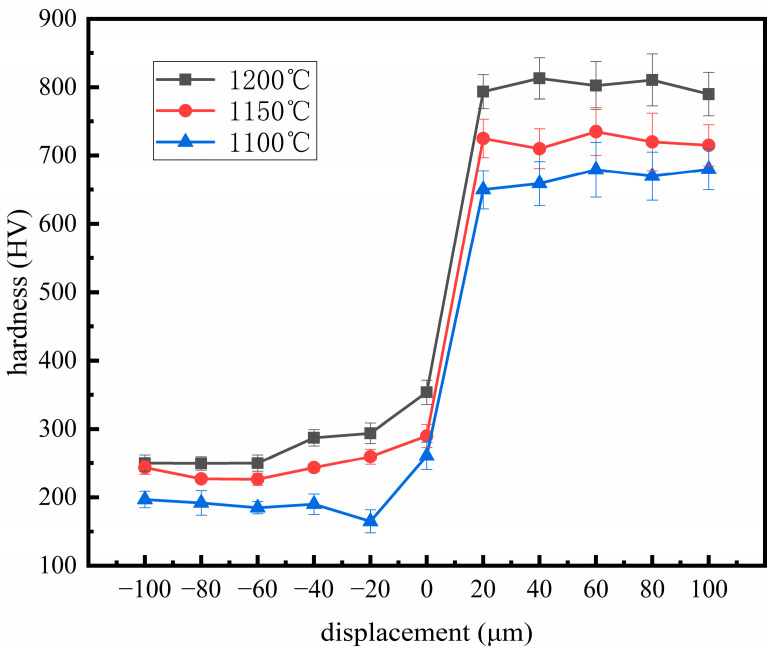
Near microhardness of HCCI powder/LCS composite plate boundary after heat treatment.

Figure 11 shows the distribution of microhardness across the cross-section of the material under different rolling temperatures. As the position shifts from the high-chromium cast iron (HCCI) side toward the low-carbon steel (LCS) side, the microhardness exhibits a gradual decrease, forming a distinct gradient. The hardness in the HCCI region remains above 800 HV, while the lowest hardness on the LCS side is approximately 185.4 ± 5.2 HV. There is a quantitative increase from 650 ± 15 HV at 1100 °C to 710 ± 12 HV at 1150 °C. At a rolling temperature of 1200 °C, the sample reaches its peak hardness in the HCCI region, with an average value of approximately 800 HV and a standard deviation of ±10 HV. This is primarily attributed to the relatively lower deformation degree at this temperature, which preserves a high volume fraction of undissolved carbides in the high-chromium cast iron, significantly enhancing the material hardness. As the rolling temperature increases, more M_7_C_3_-type carbides gradually dissolve into the austenite matrix, improving its stability. This leads to an increased content of retained austenite after subsequent quenching, resulting in an overall increase in hardness.

**Figure 12:**
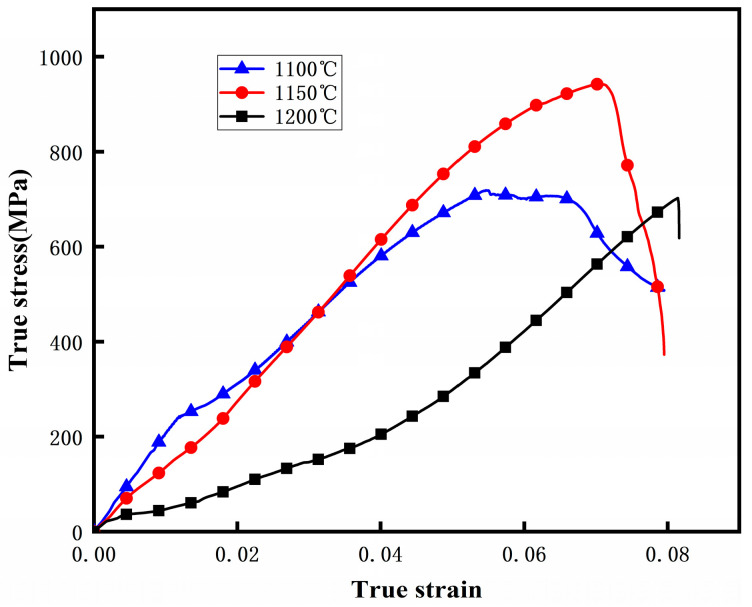
Stress–strain curves of the composite plate under different deformation temperatures.

Figure 12 presents the tensile stress–strain curves and a schematic of the maximum tensile stress of the composite plates under different rolling deformation temperatures. It can be observed that the tensile strength exhibits a non-monotonic dependence on rolling temperature: it increases from 1100 °C to 1150 °C, reaching a maximum of 810 MPa, but then decreases at 1200 °C.

The decrease in tensile strength at 1200 °C is attributed to two interrelated factors: (i) coarsening of primary carbides within the HCCI layer at excessively high temperature, which reduces the strengthening contribution from fine carbides; and (ii) over-diffusion at the interface, leading to an overly wide diffusion zone that may act as a compliant layer and promote early strain localization. These observations indicate that 1150 °C is the optimal rolling temperature for maximizing tensile strength.

In particular, the more ductile low-carbon steel layer deforms more fully, thereby more effectively coordinating the deformation of the high-chromium cast iron layer and reducing the crack sensitivity of the hard and brittle phase (high-chromium cast iron). Meanwhile, appropriate high-temperature rolling promotes interfacial bonding, reduces internal defects, and leads to more coordinated and uniform deformation of the two phases, thereby alleviating local stress concentration. The tensile stress–strain curves (Figure 12) show that specimens rolled at 1150 °C and 1200 °C exhibit slightly larger total elongation than those rolled at 1100 °C. However, this increase is primarily attributed to elastic deformation and system compliance, and does not necessarily indicate improved macroscopic plasticity. Quantitative assessment of plastic strain via residual elongation measurement is required to confirm this observation, which will be addressed in future work.

Prior to fracture, the stress continues to increase slightly with strain, indicating limited micro-plastic deformation and damage accumulation, rather than a macroscopic work-hardening stage. The specimen fractures immediately after reaching the tensile strength, with negligible uniform plastic elongation.

The tensile strength of the composite exhibits a non-monotonic dependence on the rolling temperature. As the temperature increases from 1100 °C to 1150 °C, the tensile strength improves significantly. This enhancement is primarily attributed to the enhanced plastic flow of the HCCI powder, better coordinated deformation between the two phases, and strengthened interfacial metallurgical bonding at elevated temperatures. However, a further increase in rolling temperature to 1200 °C leads to a decrease in tensile strength. This decline may be associated with excessive grain growth, the formation of brittle phases at the interface due to over-diffusion, or the initiation of micro-voids at higher processing temperatures, which ultimately compromise the load-bearing capacity.

**Figure 13:**
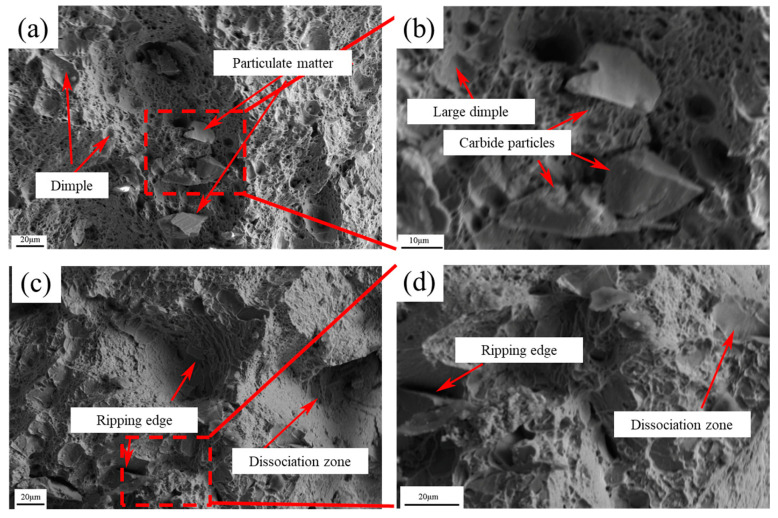
Microstructural fracture characteristics of bimetallic composite materials (**a**,**b**—1100 °C; **c**,**d**—1200 °C).

The microstructural fracture characteristics of the bimetallic composite are presented in Figure 13. The fracture surface exhibits a curved and complex propagation path, with no evident delamination between the two metals, indicating good interfacial integrity. Features such as dimples (toughening pits), large grains, and localized shear bands are observed on the fracture surface. Figure 13a,b shows cross-sectional views of the fracture surface for the sample rolled at 1100 °C, where blocky carbide spalling particles are visible. In contrast, the fracture morphology for the 1200 °C sample is shown in Figure 13c,d. Here, crack propagation tends to arrest or deflect upon encountering the tougher low-carbon steel matrix, although significant shear zones remain present. During the rolling process, the high-chromium cast iron powder is incorporated into the low-carbon steel matrix. Under applied stress, cracks typically initiate at intergranular voids or within the powder particles themselves. These cracks subsequently propagate along grain boundaries, ultimately leading to material fracture.

The obtained mechanical properties provide critical insights into the wear performance of the composites. Firstly, the high hardness (≈800 HV) of the HCCI layer (Figure 11) is the primary defense against abrasive wear, as it reduces the penetration depth and cutting action of abrasive particles. Secondly, the gradual hardness transition across the interface, as opposed to an abrupt change, indicates a metallurgically bonded and mechanically compatible interlayer. This graded structure helps mitigate stress concentration under shear forces during wear, reducing the risk of spalling or delamination of the hard layer. Thirdly, the enhanced tensile strength and interfacial bonding achieved at 1150 °C (Figure 12) directly correlate with improved wear resistance. A stronger interface ensures that the hard HCCI layer remains firmly supported by the tough steel substrate during repetitive sliding or impact, preventing premature failure. Conversely, the composite rolled at 1200 °C, despite having a similarly hard HCCI layer, exhibited lower tensile strength, suggesting a potentially weakened interface. This microstructural weakness likely served as the initiation site for crack propagation under wear stress, leading to the observed detachment of hard phase particles (as seen in Figure 8) and consequent inferior wear resistance. Therefore, optimal wear performance is not governed by hardness alone but is a synergistic result of high surface hardness, a strong and tough substrate, and most importantly, a resilient interfacial bond that maintains the integrity of the composite system under load.

## 4. Conclusions

In this study, multilayer composite billets consisting of high-chromium cast iron (HCCI) powder clad within Q235 low-carbon steel were successfully fabricated via hot rolling at different temperatures. The microstructure and dry sliding wear properties of the composites were systematically investigated, leading to the following conclusions.

1. Hot rolling at 1150 °C and 1200 °C produced dense, defect-free interfaces with continuous compositional gradients, confirming atomic-scale bonding between the HCCI powder and the LCS matrix. Nevertheless, the brittle nature of the HCCI layer prevented direct measurement of the interfacial bond strength via tensile testing. The observed fracture mode—crack initiation in HCCI followed by propagation across the interface—indirectly indicates that the interface is not the weakest link in the composite. Elemental interdiffusion, particularly of chromium, occurred across the interface to a depth of approximately 8–10 µm. Following rolling, the HCCI regions retained high levels of stored strain energy and dislocation density.
2. The material exhibited a distinct hardness gradient, decreasing progressively from the high-chromium cast iron side (hardness > 800 HV) to the low-carbon steel side (hardness ~185.4 HV). The peak hardness was attained after rolling at 1200 °C, primarily due to the retention of the highest fraction of undissolved carbides. The effect of rolling temperature on tensile strength is non-monotonic. The highest strength achieved at 1150 °C results from improved interfacial metallurgy and deformation coordination. Beyond this temperature, strength decreases, possibly due to microstructural over-aging or interface embrittlement.
3. Dry sliding wear tests indicated that abrasive wear was the dominant failure mechanism. The relatively low content of retained austenite, combined with the intrinsic brittleness of the carbides, promoted carbide spalling, thereby exacerbating wear damage.
4. Based on a comprehensive evaluation of interfacial quality, mechanical performance, and microstructural homogeneity:
  1150 °C is identified as the optimal rolling temperature for achieving the best combination of interfacial bonding, tensile strength, and hardness. This temperature provides sufficient atomic mobility for diffusion bonding while avoiding excessive carbide coarsening or interfacial degradation.
  1200 °C is suitable when maximum hardness and interfacial diffusion are prioritized, but the trade-off in tensile strength should be considered.
  1100 °C is less preferable for structural applications requiring high strength, as the interfacial bonding and densification are incomplete at this temperature.

## Figures and Tables

**Table 1:** Table of material composition (mass fraction, %).

Materials	C	Si	Cr	Mn	P	S	Ni	Fe
HCCL	3.5	1.1	26	1.0	0.02	0.03	0.9	Balance
LCS	0.15	0.1		1.5	0.01	0.002		Balance

## Data Availability

The original contributions presented in this study are included in the article. Further inquiries can be directed to the corresponding author.

## Abbreviations

The following abbreviations are used in this manuscript:

HCCI	high-chromium cast iron
LCS	low-carbon steel
